# Association between Plasma Homocysteine Concentrations and the First Ischemic Stroke in Hypertensive Patients with Obstructive Sleep Apnea: A 7-Year Retrospective Cohort Study from China

**DOI:** 10.1155/2021/9953858

**Published:** 2021-09-28

**Authors:** Nanfang Li, Xintian Cai, Qing Zhu, Xiaoguang Yao, Mengyue Lin, Lin Gan, Le Sun, Na Yue, Yingli Ren, Jing Hong, Yue Ma, Run Wang, Jina Yili, Qin Luo

**Affiliations:** ^1^Hypertension Center of People's Hospital of Xinjiang Uygur Autonomous Region, Xinjiang Hypertension Institute, National Health Committee Key Laboratory of Hypertension Clinical Research, Xinjiang Clinical Medical Research Center for Hypertension Diseases, Urumqi, Xinjiang, China; ^2^Xinjiang Medical University, Urumqi, Xinjiang, China

## Abstract

**Purpose:**

This study was aimed at investigating the association between baseline plasma homocysteine (Hcy) concentrations and the risk of the first ischemic stroke (IS) and at investigating any possible influential modifying factors in hypertensive patients with obstructive sleep apnea (OSA).

**Methods:**

Cox proportional hazards regression was employed to investigate the relationship between plasma Hcy concentration and the first IS. A generalized additive model was applied to determine the nonlinear relationship. In addition, we conducted subgroup analysis.

**Results:**

A total of 2350 hypertensive patients with OSA without a history of IS were enrolled in this study. At a median follow-up of 7.15 years, we identified 93 cases of the first IS. After adjusting for potential confounding, the findings revealed that plasma Hcy concentration was strongly and positively associated with the occurrence of the first IS (per SD increment; HR = 1.37, 95% CI: 1.30-1.44). A nonlinear relationship was found between plasma Hcy concentration and the risk of developing the first IS with inflection points for plasma Hcy of 5 *μ*mol/L. In stratified analysis, a greater positive correlation was found between baseline plasma Hcy concentrations and new-onset IS in patients with DBP ≥ 90 mmHg (per SD increment; HR = 1.48, 95% CI: 1.33-1.65 vs. <90 mmHg: HR = 1.20, 95% CI: 1.02-1.42; *P*‐interaction = 0.04) and BMI ≥ 24 and <28 kg/m^2^ (per SD increment; HR = 1.46, 95% CI: 1.26-1.70 vs. <24 kg/m^2^: HR = 1.13, 95% CI: 0.95-1.33 vs. ≥28 kg/m^2^: HR = 1.46, 95% CI: 1.25-1.70; *P*‐interaction = 0.03).

**Conclusion:**

Elevated plasma Hcy concentrations are independently associated with the risk of the first IS in hypertensive patients with OSA. Plasma Hcy concentrations ≥ 5 *μ*mol/L surely increased the risk of the first IS in hypertensive patients with OSA.

## 1. Introduction

Stroke is the leading cause of disability worldwide and the second leading cause of death after ischemic heart disease [1–3]. More importantly, according to China's National Disease Surveillance Point System, there were an estimated 2.4 million new-onset strokes and 1.1 million stroke-related deaths per year [4]. Researches have demonstrated that ischemic stroke (IS) is the most prevalent stroke subtype in China, accounting for about 70-86% of all strokes [2, 5]. Therefore, early identification and management of associated risk factors are critical measures to prevent and treat the first IS [6, 7].

Obstructive sleep apnea (OSA) is a widespread sleep disturbance [8, 9]. The high prevalence of OSA in hypertensive patients is well demonstrated, and OSA is especially prevalent in patients with intractable hypertension [10–12]. Hypertension and OSA often cooccur, which may contribute to a significantly enhanced risk of cardiovascular disease in hypertensive patients with OSA [13]. OSA is associated independently with carotid intima-media thickness in hypertensive patients [14]. OSA was additionally associated with an increased risk of new-onset ventricular fibrillation, and a significant dose-response correlation was identified between the severity of OSA and the degree of risk of developing ventricular fibrillation [15]. A meta-analysis of retrospective researches revealed that hypertensive patients with comorbid moderate to severe OSA had significantly increased mortality from cardiovascular disease compared with those without comorbid OSA [16, 17]. A few reports have indicated that people with hypertension combined with OSA have a two-fold elevated risk of developing IS compared to people without OSA [16–18].

Although previous investigations have reported a significant association between hyperhomocysteinemia (HHcy) caused by disorders of homocysteine (Hcy) metabolism and the first IS, the relationship between the degree of Hcy metabolism disorder and the first IS remains controversial [19]. In general, the normal reference range for plasma Hcy concentrations is 5 to 10 *μ*mol/L. Thus, the definition of HHcy is disputable and is commonly defined as plasma Hcy concentrations ≥ 10 *μ*mol/L, but also as Hcy ≥ 15 *μ*mol/L [20–23]. Current guidelines suggest plasma Hcy concentration control goals only in people with pure hypertension to prevent IS events [24]. However, available data from cohort investigations on the correlation between baseline plasma Hcy concentrations and incident first IS are limited and inconclusive, especially in hypertensive patients with OSA. It is worth noting that previous investigations have rarely thoroughly examined the potential moderators of the relationship between baseline plasma Hcy concentrations and incident first IS risk.

Therefore, we intended to evaluate the association between baseline plasma Hcy concentrations and the first IS and to investigate the appropriate plasma Hcy concentration target to reduce the incidence of the first IS in hypertensive patients with OSA.

## 2. Materials and Methods

### 2.1. Study Design

This retrospective cohort study included consecutive hypertensive patients with suspected OSA hospitalized at the Hypertension Center of People's Hospital of Xinjiang Uygur Autonomous Region from January 2011 to December 2013. The study protocol was authorized by the Medical Ethics Committee of the People's Hospital of Xinjiang Uygur Autonomous Region (no. 2019030662) and was conducted in adherence to the approved guidelines. Because of the retrospective nature of the study, patient consent and/or informed consent was not required.

A total of 2585 patients aged ≥18 years with hypertension combined with OSA determined by polysomnography (PSG) were enrolled in this study. Exclusion criteria for this study were age younger than 18 years (*n* = 11), severe systemic disease (*n* = 25) (i.e., severe pulmonary disease, malignancy, severe liver disease, or severe chronic kidney disease or other major diseases that affect long-term survival), central sleep apnea (*n* = 12), pregnancy (*n* = 6), lacking a fasting blood sample and physical examination at baseline (*n* = 61), a history of stroke at baseline (*n* = 24), and loss to follow-up (*n* = 96). Ultimately, 2350 study participants were eligible for inclusion in the statistical analysis.

### 2.2. Baseline Examination

All participants finished the baseline examination between January 2011 and December 2013. Data on demographic features, lifestyle, personal disease records, history of regular CPAP treatment, and medication history were collected from all candidates through interviews.

Weight, height, neck circumference (NC), and waist circumference (WC) were measured three times, and the mean of the three times measurements was used for analysis. Systolic blood pressure (SBP) and diastolic blood pressure (DBP) were monitored using an electronic sphygmomanometer (HEM-1000, Omron, Kyoto, Japan). All participants underwent PSG examination throughout the night. All participants were instructed to refrain from coffee, alcohol, and sedative-hypnotics before the sleep study. Interpretation of PSG results was done by a professional polysomnography technician, and all steps were consistent with previous studies [25, 26]. Venous blood samples were retrieved from the antecubital vein in the early morning after 12 hours of fasting. Fasting plasma glucose (FPG), high-density lipoprotein (HDL-c), total cholesterol (TC), low-density lipoprotein (LDL-c), triglycerides (TG), creatinine (Cr), and high-sensitivity C-reactive protein (hs-CRP) levels were measured. Plasma Hcy concentrations were measured by a fluorescence polarization immunoassay on an automated immunoassay analyzer. The estimated glomerular filtration rate (eGFR) was calculated by using a formula derived from the CKD-EPI [27].

### 2.3. Definitions

The apnea hypopnea index (AHI) was defined as the sum of apnea and hypopnea events per hour of sleep on average. A diagnosis of OSA was determined as a minimum of 5 events per hour of AHI, with 5 to 14.9 events per hour recognized as mild OSA, 15 to 29.9 events per hour as moderate OSA, and 30 or more events per hour as severe OSA. Hypertension was defined as SBP ≥ 140 mmHg and/or DBP ≥ 90 mmHg and/or previously diagnosed hypertension and/or use of antihypertensive therapy within the past two weeks. Patients with diabetes were defined as those previously diagnosed with diabetes or newly diagnosed with diabetes (fasting glucose ≥ 7.0 mmol/L on 3 occasions during hospitalization, HbA1c > 6.5% at baseline), according to current guidelines. According to the frequency of drinking and smoking, we categorized them as never, former, and current.

### 2.4. Clinical Outcomes and Follow-Up

Endpoints were acquired through personal interview, records from medical insurance, and hospital discharge summaries. According to former investigations, the principal endpoint was to be determined as the first IS, including cerebral infarction and transient ischemic attack [28, 29]. The diagnosis of IS was on the basis of a contract vascular computed tomography (CT) scan or cranial CT scan, cerebrovascular angiography, or magnetic resonance imaging of the brain. The period of follow-up started at the first visit and ended on December 31, 2020.

### 2.5. Statistical Analysis

Differences between baseline characteristics of the different plasma Hcy groups were analyzed by one-way ANOVA, Kruskal-Wallis *H*, and *χ*^2^ tests. Multivariate Cox proportional hazard regression was performed to evaluate the association between baseline plasma Hcy concentration and the risk of the first IS by estimating hazard ratios (HR) and 95% confidence intervals (CI). Adjustment for variables in this study showed varying degrees of adjustment results according to the statement of STROBE [30]. Adjust model I was adjusted for age and gender at baseline; adjusted model II was further adjusted for smoking status, drinking status, NC, BMI, and WC at baseline; adjusted model III was further adjusted for history of arrhythmia, diabetes, coronary heart disease, SBP, DBP, AHI, AI, HI, sleep duration, mean SaO_2_ and lowest SaO_2_, antidiabetic drugs, lipid-lowering drugs, antiplatelet drugs, regular CPAP treatment, antihypertensive drugs, and TC, TG, HDL-c, LDL-c, FPG, eGFR, Cr, and hs-CRP levels at baseline. Moreover, we calculated the cumulative IS incidence function of events over time using the Kaplan-Meier method. Further, we simulated the dose-response correlation between plasma Hcy concentration and IS risk using a generalized additive model, fitted the model with a recursive algorithm using maximum likelihood, and calculated the inflection points for the nonlinear correlation [31]. Considering that association between plasma Hcy concentrations and IS may differ in some populations, we performed exploratory stratified analysis by using a Cox proportional hazards model for some subgroups and used likelihood ratio tests to check for hierarchical differences to determine if there was an interaction. Finally, to estimate the reasonableness of the deviations caused by unmeasured and residual confounding factors, we also calculated the *E*-value of our main research results. The *E*-value estimates the strength of the unmeasured confounding variable needed to invalidate the observed association between our exposure and the result, taking into account all the measured covariables.

All statistical analyses were conducted using R version 4.0.1 software.

## 3. Results

### 3.1. Demographic Characteristics

**Figure 1:**
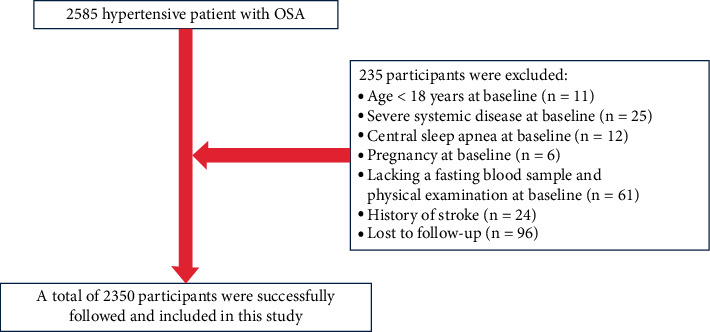
Flowchart.

In this cohort study, a total of 2350 participants, including 1611 males and 739 females, were evaluated. Participant screening details are shown in Figure 1.  Table 1 shows the baseline characteristics of the participants grouped by plasma Hcy concentration tertile. Overall, the mean age of the 2350 participants was 49.45 ± 10.65 years, and 68.55% were male.

### 3.2. Follow-Up Results

**Figure 2:**
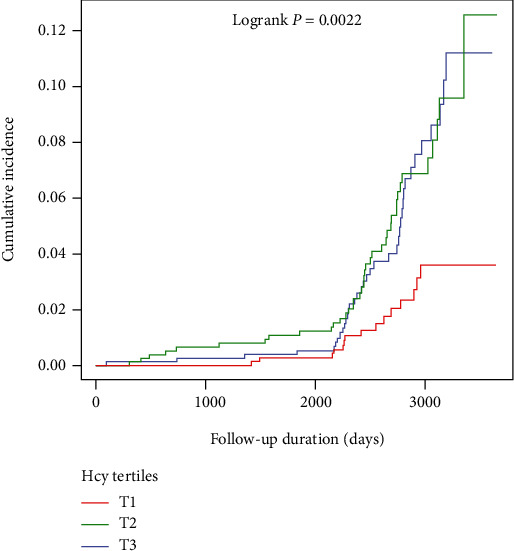
Kaplan-Meier curves of incidence of ischemic stroke according to tertiles of baseline plasma Hcy concentrations.

During follow-up, 93 (3.96%) of the hypertensive patients with OSA in the study were diagnosed with IS. Of these, the incidence of IS corresponding to the plasma Hcy concentrations tertile grouping was 1.92% for T1, 4.99% for T2, and 4.71% for T3.  Figure 2 illustrates the significant difference in IS risk between plasma Hcy concentration tertile groups (log-rank test, *P* = 0.0022). The cumulative risk of IS gradually increased with increasing plasma Hcy concentrations.

### 3.3. Association between Plasma Hcy Concentrations and the First IS in Hypertensive Patients with OSA

The association between plasma Hcy concentrations and the first IS is summarized in Table 2. We found that high plasma Hcy concentrations, expressed as a continuous variable and a categorical variable, were significantly and positively associated with the first IS. When plasma Hcy concentrations were expressed as a continuous variable, plasma Hcy concentrations were significantly associated with first IS in the crude model (per SD increment; HR = 1.37, 95% CI: 1.30-1.44, *P* < 0.01; *E*‐value = 2.08). In adjust model I, plasma Hcy concentrations were still an independent risk factor for the first IS (per SD increment; HR = 1.35, 95% CI: 1.27-1.42, *P* < 0.01; *E*‐value = 2.03). In adjust model II, plasma Hcy concentrations were still an independent risk factor for the first IS (per SD increment; HR = 1.34, 95% CI: 1.26-1.43, *P* < 0.01; *E*‐value = 2.01), as well as the fully adjusted model (adjust model III), where plasma Hcy concentrations (per SD increment; HR = 1.32, 95% CI: 1.24-1.38, *P* < 0.01; *E*‐value = 1.97) were positively associated with the first occurrence of IS (Table 2). In the sensitivity analysis, all *E*-values were more than one, indicating that the current association tends to be stable and that our main findings are unlikely to be offset by unmeasured confounding variables. Moreover, when plasma Hcy concentrations were divided into different categories, there were obvious changes in effect sizes. The risk of the first IS increased over time by baseline plasma Hcy concentration tertiles and was still significant despite adjustment for potential confounders, and the fully adjusted HRs (adjusted model III) were 2.13 (95% CI: 1.74-2.59) and 1.76 (95% CI: 1.43-2.17) for tertiles 2 and 3, respectively, versus tertile 1 of the baseline plasma Hcy concentrations.

### 3.4. Threshold Effect Analysis of Plasma Hcy Concentrations on Incident First IS

**Figure 3:**
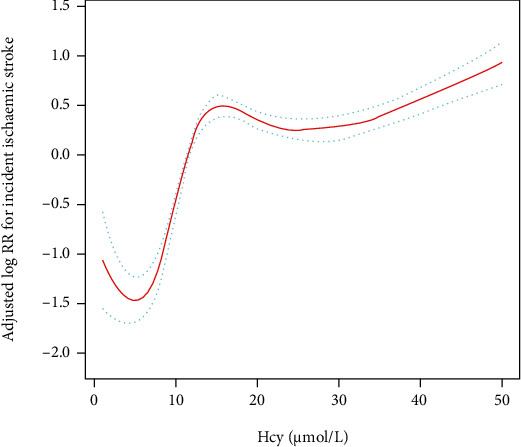
A piecewise linear regression model was used to detect the association of plasma Hcy concentrations and the first IS according to the plasma Hcy concentration cut points. All were adjusted for age, gender, smoking status, drinking status, NC, WC, BMI, SBP, DBP, history of arrhythmia, diabetes, AHI, AI, HI, sleep duration, mean SaO_2_ and lowest SaO_2_, antidiabetic drugs, antiplatelet drugs, lipid-lowering drugs, regular CPAP treatment, antihypertensive drugs, and TC, TG, HDL-c, LDL-c, FPG, eGFR, Cr, and hs-CRP levels at baseline.

After adjusting for potential confounders, a nonlinear relationship was observed between plasma Hcy concentration and the first IS (Figure 3). The inflection point, determined by the two-piecewise recursive algorithm and linear regression, was 5 *μ*mol/L. The *P* value of the log-likelihood ratio test was less than 0.01, indicating that the two-piecewise linear regression was more appropriate for fitting the relationship between plasma Hcy concentrations and the risk of the first IS. On the right of the inflection point (plasma Hcy concentrations ≥ 5 *μ*mol/L), we observed a positive association between plasma Hcy concentrations and the occurrence of the first IS (HR = 1.35, 95% CI: 1.08-1.69, *P* < 0.01). On the left side of the inflection point (plasma Hcy concentrations < 5 *μ*mol/L), however, their relationship saturated (HR = 1.02, 95% CI: 0.97-1.07, *P* = 0.28) (Table 3).

### 3.5. Stratified Analyses

To better identify plasma Hcy concentrations and other possible influences on the risk of the first IS, we performed stratified analyses and interaction tests in prespecified subgroups. In stratified analyses, a greater positive correlation between baseline plasma Hcy concentrations and new-onset IS was observed for participants with DBP ≥ 90 mmHg (per SD increment; HR = 1.48, 95% CI: 1.33-1.65 vs. <90 mmHg: HR = 1.20, 95% CI: 1.02-1.42, *P*‐interaction = 0.04) and BMI ≥ 24 and <28 kg/m^2^ (per SD increment; HR = 1.46, 95% CI: 1.26-1.70 vs. <24 kg/m^2^: HR = 1.13, 95% CI: 0.95-1.33 vs. ≥28 kg/m^2^: HR = 1.46, 95% CI: 1.25-1.70, *P*‐interaction = 0.03) (Table 4). However, other variables, including gender (female vs. male), diabetes (no vs. yes), smoking status (never vs. ever vs. current), arrhythmia (no vs. yes), drinking status (never vs. ever vs. current), age (<60 vs. ≥60 years), FPG (<6.1 vs. ≥6.1 mmol/L), SBP (<140 vs. ≥140 mmHg), AHI (≥5 and <15 vs. ≥15 and <30 vs. ≥30 events/hour), and eGFR (<90 vs. ≥90 mL/min/1.73 m^2^) at baseline, did not dramatically modify the relationship between plasma Hcy concentrations and the first IS (all *P*‐interactions > 0.05) (Table 4).

## 4. Discussion

In China, IS is the most prevalent type of cerebrovascular event. Therefore, the prevention of IS is an important and urgent public health issue [6, 7, 32]. Since the discovery of the association of Hcy with the pathogenesis of atherosclerosis, Hcy-lowering therapies have attracted considerable attention among the various prevention strategies for IS [33, 34]. In addition, a compelling and developing body of epidemiological evidence demonstrates a significant correlation between increased Hcy concentrations and enhanced risk of IS [35, 36]. However, the relationship between Hcy and IS is inconclusive. Iso et al. conducted a prospective, nested case-control study of 11846 Japanese subjects aged 40 to 85 years. Their findings showed odds ratios (95% CI) of 3.89 (1.60 to 9.46) for the highest (≥11.0 *μ*mol/L) versus the lowest quartile (<7.0 *μ*mol/L) of Hcy for IS after adjusting for cardiovascular risk factors. The respective odds ratio associated with a 5 *μ*mol/L increase in Hcy was 1.52 (1.07 to 2.14) [37]. Sacco et al. followed a population-based cohort for vascular events. Their findings suggest that Hcy elevations above 15 *μ*mol/L are an independent risk factor for IS, whereas mild tHcy elevations of 10 to 15 *μ*mol/L are not predictive. Hcy has the greatest vascular impact in Whites and Hispanics and less in Blacks [38]. However, the Caerphilly study conducted by Fallon et al. yielded the opposite results to the above study. A total of 2254 male participants aged 50 to 64 years were included in the Caerphilly study, and after a mean follow-up of 10.2 years, a total of 107 participants experienced IS. However, after adjusting for confounding, no significant association was found between Hcy and IS [39].

In the current study, we found a meaningful relationship between plasma Hcy concentrations and the incidence of the first IS in hypertensive patients with OSA. Similar results have been reported in several previous studies in patients with simple hypertension or healthy populations, but these studies did not identify a nonlinear association [38, 40, 41]. We found a threshold saturation effect association between plasma Hcy concentrations and the first IS even after removing adjusted covariates from the model. This is the first study to explore the nonlinear association between plasma Hcy concentration and the first IS, calculated for plasma Hcy concentrations at the inflection points of 5 and 15 *μ*mol/L. Notably, this association between plasma Hcy concentrations and the first IS has opposite effects in the middle of the two inflection points versus the left and right sides. Plasma Hcy concentrations were positively associated with the risk of the first IS when plasma Hcy concentrations were between 5 and 15 *μ*mol/L. This suggests that the risk of the first IS in hypertensive patients with OSA increases rapidly when plasma Hcy concentrations are between 5 and 15 *μ*mol/L. In contrast, there was no significant association between plasma Hcy concentrations and the risk of the first IS when plasma Hcy concentrations were <5 *μ*mol/L or >15 *μ*mol/L. However, the mechanism behind the threshold saturation effect association between plasma Hcy concentrations and the first IS and the inflection point is not clear. To better understand the association between plasma Hcy concentrations and the risk of the first IS, we included significant variables in univariate analysis and noncollinear variables in the multivariate analysis. After adjustment for covariates, participants in the highest tertile of plasma Hcy concentrations had a 1.76-fold greater risk of the first IS compared with those in the lowest tertile.

Subgroup analysis and exploration of interactions are essential for clinical research to better understand plasma Hcy concentrations and the risk of the first IS in different populations. In this study, these factors, including gender, diabetes, coronary heart disease, arrhythmia, smoking status, drinking status, age, BMI, FPG, SBP, DBP, AHI, and eGFR, and stronger correlations were identified in participants with BMI ≥ 24 kg/m^2^ and DBP ≥ 90 mmHg. In China, the prevalence of obesity in hypertensive patients with OSA is high [42]. A meta-analysis identified a 40% increase in IS mortality for every 5 kg/m^2^ increase in BMI among those with a BMI between 25 and 50 kg/m^2^. However, there was no association between BMI and IS mortality in those with a BMI of 15-24 kg/m^2^ [43]. The exact biological mechanism of the interaction between high BMI and high Hcy is unclear. In available studies, a reasonable biological interpretation of this interaction may be due to the fact that elevated Hcy and obesity may share several cellular and molecular mechanisms (e.g., impaired mitochondrial function and the onset of oxidative stress) that are responsible for the development of stroke [44, 45]. In addition, we found that baseline DBP (≥90 mmHg vs. <90 mmHg) significantly altered the effect of Hcy on the risk of the first IS. Zheng et al. [46] found that in Chinese patients with uncontrolled hypertension, the prevalence of IS increased with increasing DBP, suggesting that DBP has an important contribution to the incidence of IS in hypertensive patients. This suggests that DBP serves an essential role in the incidence of IS in hypertensive patients. DBP is traditionally thought to reflect structural changes in small arteries or thinning of microvessels. Thus, elevated DBP may reflect dysfunction of peripheral microvessels, whereas elevated Hcy reflects damage to large- and medium-sized arteries; thus, elevated levels of Hcy and DBP may reflect damage to peripheral vessels and large- and medium-sized arteries, which helps explain the apparent concerted impact of DBP and Hcy on the risk of the first IS [47, 48]. The optimal blood pressure level for controlling Hcy on the risk of the first IS should be explored in future studies.

Our findings may have important implications for public health concerns [19, 49]. In hypertensive patients with OSA, increased Hcy levels are probably a changeable risk factor for IS. Lowering Hcy levels may reduce the risk of stroke in hypertensive patients with OSA, and the prognostic effect of Hcy levels has important clinical implications. However, the relationship between slightly increased Hcy levels and the risk of IS is disputable [38]. Our study revealed a nonlinear dose-response relationship between Hcy levels and the first IS in hypertensive patients with OSA, and slightly increased Hcy levels may increase the risk of the first IS in hypertensive patients with OSA. However, a few investigations have shown that slightly increased Hcy levels do not enhance the risk of IS [38, 50]. Further, in IS patients with marginal or slightly increased Hcy levels, reducing Hcy with high-dose vitamin therapy did not reduce the risk of IS recurrence [51]. Therefore, additional investigations are necessary to verify whether the risk of IS in hypertensive patients with OSA can be reduced by intensive control or maintenance of lower Hcy levels through folic acid and vitamin B12 supplementation.

The present study has some unique strengths. First, it is the first report of a nonlinear relationship between Hcy and the first IS in hypertensive patients with OSA. Second, the present study is a retrospective cohort research and thus vulnerable to potential influences. However, we applied rigorous statistical adjustments to reduce remnant confounders as much as possible. Finally, we conducted subgroup analyses and interaction tests to further demonstrate reliability for the results and to identify potential interactions with other variables.

Limitations of the study are mainly in the areas of the following. First, levels of Hcy were tested only once at baseline, so we were unable to investigate the effect of changes in levels of Hcy on IS. Second, levels of Hcy could be affected by genetic background, dietary habits, and/or medication usage. It is possible that a single plasma Hcy concentration test may not provide us with sufficient information to determine a causal relationship between Hcy and IS. Third, participants were limited to hypertensive patients with OSA in northwest China. Therefore, the findings of the present investigation should be approached with caution for extrapolation to the general population or other ethnic communities. Fourth, data on adiponectin, IL-6, and TNF-*α* levels are lacking in this cohort study, so it is not possible to compare the accuracy of Hcy levels and other biomarkers for predicting the risk of IS [2, 5]. Finally, recall bias may have existed during data collection. However, recall bias was minimized during data collection through rigorous training in survey methodology and the application of standard operating procedures.

## 5. Conclusion

In conclusion, elevated plasma Hcy concentrations were independently associated with the risk of the first IS in hypertensive patients with OSA. Plasma Hcy concentrations ≥ 5 *μ*mol/L obviously increase the risk of the first IS in hypertensive patients with OSA. Undoubtedly, lowering plasma Hcy concentrations to <5 *μ*mol/L is an efficient and modest approach to minimize the risk of the first IS in hypertensive patients with OSA.

## Figures and Tables

**Table 1:** Participants sorted by tertiles of Hcy.

Variable	Hcy tertiles (mmol/L)			*P* value
	Tertile 1 (<11.6)	Tertile 2 (11.6–18.2)	Tertile 3 (>18.2)	
No. of participants	783	782	785	
Age (years)	48.87 ± 9.94	50.36 ± 10.59	49.15 ± 11.29	0.01
BMI (kg/m^2^)	28.46 ± 3.78	28.43 ± 3.89	28.46 ± 3.79	0.98
NC (cm)	40.04 ± 3.89	40.38 ± 3.93	40.76 ± 3.60	<0.01
WC (cm)	100.66 ± 10.06	101.24 ± 10.74	101.11 ± 10.30	0.51
SBP (mmHg)	138.52 ± 18.66	139.65 ± 18.89	142.19 ± 21.28	<0.01
DBP (mmHg)	91.10 ± 13.93	91.04 ± 13.08	93.63 ± 15.30	<0.01
Gender (*n* (%))				<0.01
Female	293 (37.42%)	264 (33.76%)	179 (22.80%)	
Male	490 (62.58%)	518 (66.24%)	606 (77.20%)	
Smoking status (*n* (%))				<0.01
Never	484 (61.81%)	463 (59.21%)	393 (50.06%)	
Former	72 (9.20%)	80 (10.23%)	97 (12.36%)	
Current	227 (28.99%)	239 (30.56%)	295 (37.58%)	
Drinking status (*n* (%))				0.10
Never	528 (67.43%)	527 (67.90%)	502 (63.95%)	
Former	39 (4.98%)	54 (6.91%)	63 (8.03%)	
Current	216 (27.59%)	197 (25.19%)	220 (28.03%)	
History of disease				
Arrhythmia (*n* (%))	109 (13.92%)	178 (22.76%)	153 (19.49%)	<0.01
Diabetes (*n* (%))	151 (19.28%)	150 (18.77%)	115 (14.65%)	0.06
Laboratory examinations				
TC (mmol/L)	4.61 ± 1.31	4.47 ± 0.95	4.59 ± 1.34	0.06
TG (mmol/L)	1.77 (1.28-2.47)	1.73 (1.22-2.48)	1.76 (1.25-2.54)	0.25
HDL-c (mmol/L)	1.11 ± 0.29	1.11 ± 0.31	1.09 ± 0.28	0.32
LDL-c (mmol/L)	2.61 ± 0.81	2.72 ± 0.80	2.63 ± 0.81	0.02
hs-CRP (mg/L)	2.21 (0.90-4.07)	2.01 (0.96-4.19)	2.12 (0.99-3.83)	0.85
FPG (mmol/L)	5.30 ± 1.35	5.33 ± 1.59	5.21 ± 1.48	0.26
Hcy (*μ*mol/L)	7.70 ± 3.32	14.70 ± 1.87	29.22 ± 10.15	<0.01
eGFR (mL/min/1.73 m^2^)	99.26 ± 21.08	95.75 ± 20.50	93.39 ± 22.11	<0.01
Cr (*μ*mol/L)	73.60 ± 24.55	76.21 ± 20.98	81.62 ± 25.22	<0.01
Polysomnography examinations				
AHI (events/hour)	17.40 (10.00-33.80)	19.40 (10.40-34.95)	18.50 (10.50-32.80)	0.52
AI (events/hour)	2.50 (0.40-9.80)	3.20 (0.50-12.28)	3.10 (0.60-11.30)	0.79
HI (events/hour)	12.40 (7.20-21.40)	12.85 (7.30-21.17)	12.10 (7.20-19.20)	0.10
Sleep duration (minutes)	368.17 ± 73.55	366.11 ± 73.24	367.05 ± 72.27	0.86
Mean SaO_2_ (%)	90.83 ± 9.62	91.35 ± 6.86	91.16 ± 8.01	0.46
Lowest SaO_2_ (%)	76.54 ± 12.32	77.12 ± 10.00	77.51 ± 11.04	0.23
Medication use				
Antidiabetic drugs (*n* (%))	133 (16.99%)	79 (10.10%)	102 (12.99%)	<0.01
Lipid-lowering drugs (*n* (%))	417 (53.26%)	536 (68.54%)	605 (77.07%)	<0.01
Antihypertensive drugs (*n* (%))	713 (91.06%)	737 (94.25%)	766 (97.58%)	<0.01
Antiplatelet drugs (*n* (%))	541 (69.09%)	620 (79.28%)	508 (64.71%)	<0.01
Regular CPAP treatment (*n* (%))	22 (3.07%)	41 (6.27%)	34 (2.04%)	<0.01
Follow-up duration (days)	2627.00 (2313.00-2995.00)	2655.00 (2287.50-2984.75)	2563.00 (2265.00-2973.00)	0.26
Incident ischemic stroke (*n* (%))				<0.01
No	768 (98.08%)	743 (95.01%)	748 (95.29%)	
Yes	15 (1.92%)	39 (4.99%)	37 (4.71%)	

Data are *n* (%), mean ± SD, or median (interquartile range). BMI: body mass index; NC: neck circumference; WC: waist circumference; SBP: systolic blood pressure; DBP: diastolic blood pressure; TC: total cholesterol; TG: triglyceride; HDL-c: high-density lipoprotein cholesterol; LDL-c: low-density lipoprotein cholesterol; hs-CRP: high-sensitivity C-reactive protein; FPG: fasting plasma glucose; Hcy: homocysteine; eGFR: estimated glomerular filtration rate; Cr: creatinine; AHI: apnea hypopnea index; AI: apnea index; HI: hypopnea index; mean SaO_2_: mean oxygen saturation; lowest SaO_2_: lowest oxygen saturation; CPAP: continuous positive airway pressure.

**Table 2:** Association between Hcy and incident of the first IS in different models.

Exposure	Crude model (HR to 95% CI to *P*)	Adjust model I (HR to 95% CI to *P*)	Adjust model II (HR to 95% CI to *P*)	Adjust model III (HR to 95% CI to *P*)
Continuous				
Hcy (per SD increment)	1.37 (1.30 to 1.44) <0.01	1.35 (1.27 to 1.42) <0.01	1.34 (1.26 to 1.43) <0.01	1.32 (1.24 to 1.38) <0.01
Categorical				
Hcy tertiles (*μ*mol/L)				
Tertile 1 (<11.6)	Reference	Reference	Reference	Reference
Tertile 2 (11.6–18.2)	2.91 (2.42 to 3.49) <0.01	2.67 (2.22 to 3.20) <0.01	2.33 (1.94 to 2.72) <0.01	2.13 (1.74 to 2.59) <0.01
Tertile 3 (>18.2)	2.75 (2.29 to 3.31) <0.01	2.23 (1.85 to 2.70) <0.01	1.96 (1.73 to 2.19) <0.01	1.76 (1.43 to 2.17) <0.01

Crude model: adjusted for none. Adjust model I: adjusted for age and gender at baseline. Adjust model II: adjusted for variables in adjusted model I plus smoking status, drinking status, NC, WC, and BMI at baseline. Adjust model III: fully adjusted model. Adjusted for variables in adjusted model II plus SBP, DBP, history of arrhythmia and diabetes, AHI, AI, HI, sleep duration, mean SaO_2_ and lowest SaO_2_, antidiabetic drugs, antiplatelet drugs, lipid-lowering drugs, regular CPAP treatment, antihypertensive drugs, and TC, TG, HDL-c, LDL-c, FPG, eGFR, Cr, and hs-CRP levels at baseline. Abbreviations are the same as in Table 1.

**Table 3:** A piecewise linear regression model was applied to detect the association of Hcy and IS according to the Hcy cut points.

Outcome: incident of ischemic stroke	HR (95% CI)	*P* value
Linear regression	1.13 (1.02 to 1.24)	<0.01
Two-piecewise linear regression model		
Hcy < 5 *μ*mol/L	1.02 (0.97 to 1.07)	0.28
Hcy ≥ 5 *μ*mol/L	1.35 (1.08 to 1.69)	<0.01
*P* for the log-likelihood ratio test	<0.01	

Notes: adjusted for age, gender, smoking status, drinking status, NC, WC, BMI, SBP, DBP, history of arrhythmia, diabetes, AHI, AI, HI, sleep duration, mean SaO2 and lowest SaO2, antidiabetic drugs, antiplatelet drugs, lipid-lowering drugs, regular CPAP treatment, antihypertensive drugs, and TC, TG, HDL-c, LDL-c, FPG, eGFR, Cr, and hs-CRP levels at baseline. Abbreviations are the same as in Table 1.

**Table 4:** Association between Hcy and the first IS in various subgroups.

Stratification variable	No. of participants.	HR (95% CI)	95% CI low	95% CI high	*P* value	*P* for interaction
Gender						0.63
Female	744	1.20	0.65	2.22	0.56	
Male	1606	1.40	1.13	1.74	<0.01	
Diabetes						0.51
No	1937	1.39	1.12	1.73	<0.01	
Yes	413	1.16	0.70	1.92	0.57	
Arrhythmia						0.18
No	2306	1.24	1.05	1.46	0.01	
Yes	440	1.41	1.27	1.57	<0.01	
Smoking status						0.63
Never	1342	1.33	0.94	1.88	0.10	
Former	258	1.78	1.04	3.05	0.04	
Current	750	1.34	0.99	1.80	0.05	
Drinking status						0.65
Never	1557	1.43	1.12	1.82	<0.01	
Former	166	1.24	0.63	2.46	0.54	
Current	627	1.13	0.72	1.79	0.59	
Age (years)						0.87
<60	1911	1.36	1.07	1.73	0.01	
≥60	439	1.42	0.96	2.08	0.08	
BMI (kg/m^2^)						0.03
<24	221	1.13	0.95	1.33	0.16	
≥24, <28	934	1.46	1.26	1.70	<0.01	
≥28	1195	1.46	1.25	1.70	<0.01	
SBP (mmHg)						0.41
<140	1084	1.25	0.86	1.82	0.23	
≥140	1266	1.51	1.18	1.92	<0.01	
DBP (mmHg)						0.04
<90	887	1.20	1.02	1.42	0.03	
≥90	1463	1.48	1.33	1.65	<0.01	
AHI (events/hour)						0.37
≥5, <15	934	1.46	1.04	2.07	0.03	
≥15, <30	722	1.47	1.04	2.09	0.03	
≥30	694	1.07	0.73	1.56	0.74	
FPG (mmol/L)						0.72
<6.1	1991	1.38	1.23	1.54	<0.01	
≥6.1	359	1.32	1.04	1.66	0.02	
eGFR (mL/min/1.73 m^2^)						0.59
<90	941	1.42	1.08	1.85	0.01	
≥90	1409	1.27	0.93	1.72	0.13	

Note 1: adjusted for age, gender, smoking status, drinking status, NC, WC, BMI, SBP, DBP, history of arrhythmia, diabetes, AHI, AI, HI, sleep duration, mean SaO_2_ and lowest SaO_2_, antidiabetic drugs, antiplatelet drugs, lipid-lowering drugs, regular CPAP treatment, antihypertensive drugs, and TC, TG, HDL-c, LDL-c, FPG, eGFR, Cr, and hs-CRP levels at baseline. Note 2: in each case, the model was not adjusted for hierarchical covariates. Abbreviations are the same as in Table 1.

## Data Availability

All relative data are in the paper.
